# FluoroCellTrack: An algorithm for automated analysis of high-throughput droplet microfluidic data

**DOI:** 10.1371/journal.pone.0215337

**Published:** 2019-05-01

**Authors:** Manibarathi Vaithiyanathan, Nora Safa, Adam T. Melvin

**Affiliations:** Cain Department of Chemical Engineering, Louisiana State University, Baton Rouge, Louisiana, United States of America; Texas A&M University College Station, UNITED STATES

## Abstract

High-throughput droplet microfluidic devices with fluorescence detection systems provide several advantages over conventional end-point cytometric techniques due to their ability to isolate single cells and investigate complex intracellular dynamics. While there have been significant advances in the field of experimental droplet microfluidics, the development of complementary software tools has lagged. Existing quantification tools have limitations including interdependent hardware platforms or challenges analyzing a wide range of high-throughput droplet microfluidic data using a single algorithm. To address these issues, an all-in-one Python algorithm called FluoroCellTrack was developed and its wide-range utility was tested on three different applications including quantification of cellular response to drugs, droplet tracking, and intracellular fluorescence. The algorithm imports all images collected using bright field and fluorescence microscopy and analyzes them to extract useful information. Two parallel steps are performed where droplets are detected using a mathematical Circular Hough Transform (CHT) while single cells (or other contours) are detected by a series of steps defining respective color boundaries involving edge detection, dilation, and erosion. These feature detection steps are strengthened by segmentation and radius/area thresholding for precise detection and removal of false positives. Individually detected droplet and contour center maps are overlaid to obtain encapsulation information for further analyses. FluoroCellTrack demonstrates an average of a ~92–99% similarity with manual analysis and exhibits a significant reduction in analysis time of 30 min to analyze an entire cohort compared to 20 h required for manual quantification.

## Introduction

Development of fluorescence and image-based single cell technologies has enabled systematic investigation of cellular heterogeneity in a wide range of diseased tissues and cellular populations [1, 2]. While conventional single cell analytical tools like flow cytometry (and Fluorescence Activated Cell Sorting, Image Flow Cytometry) can detect, sort and collect cells with desired properties, these techniques do not permit dynamic monitoring of cell responses as the data is collected at a single time point [3]. Considering these limitations, microscale technologies such as droplet microfluidic devices and microfluidic cell trap arrays allow for facile collection and segregation of single cells to enable real-time investigation of cellular processes [4, 5]. Droplet microfluidic devices in particular, have an advantage of working with picoliter to nanoliter volumes of solution that increases sensitivity, specificity, and precise quantification of real-time intra and extracellular processes [3]. The development of a wide variety of sophisticated cellular fluorescent probes in recent times has enabled easy tracking and detection of cellular activities by incorporating static microdroplet trapping arrays with fluorescence microscopy platforms to eliminate the need for high-speed cameras and expensive fiber optics used in large-scale cytometric tools [6, 7]. This technology has found a diverse set of applications in disease detection and diagnostics ranging from single cell analyses to droplet-based quantitative PCR and electrokinetic assays [8–11]. One such example in cellomics is the use of fluorescent stains and organic dyes in droplet microfluidic devices to sort cells based on their dynamic fluorescent responses to external stimuli [12, 13]. Similarly, fluorescent proteins, quantum dots, and luminescent nanoparticles have been used to track protein-protein interactions, intracellular enzyme activities, and identify biomolecules or biomarkers within single cells encapsulated in droplets [14–17]. In addition to cellomics, massively parallelized high-throughput droplet generators are used in combination with fluorescent barcodes to perform single cell DNA- and RNA- sequencing [18, 19]. Digital droplet microfluidics are also extensively used in the quantitative immunoassays and development of biosensors [20]. Beyond disease detection and diagnostics, fluorescence-based droplet microfluidics also finds applications in renewable energy, pharmaceutical industry and managing environmental issues [21–24].

The growing advancement of these single-cell analytical devices in various fields has created a need for specific computational tools capable of processing and quantifying the large amount of intricate data collected from these screening systems. Additionally, there is a need for automated quantification due to the inconsistencies and challenges associated with manual cell counting and analysis, which is highly laborious and prone to subjective results that may vary from person to person. While many of the end-point commercial single cell analysis platforms are equipped with expensive inter-complementary hardware and software options to perform simultaneous imaging and analysis, there are limited stand-alone software tools to analyze high-throughput microarray or microfluidics data. Existing open-source software like ImageJ (NIH) and CellProfiler (Broad Institute) can count and characterize the total number of cells in a defined region; however, this is not suitable for high-throughput intracellular quantification of a large number of cells such as the datasets generated using a droplet microfluidic trapping array [25, 26]. ImageJ utilizes macros to provide grid analysis similar to a hemocytometer but is limited to grid annotation or the detection cell colonies [27]. Commercial platforms accompanied by software tools like WOLF Cell Sorter (NanoCellect Biomedical, Inc., San Diego, CA, USA), Scepter 2.0 Cell Counter (Millipore Sigma, Burlington, MA, USA) and Cellomics (ThermoScientific, Pittsburg, PA, USA) can analyze high-throughput microfluidic data, but are limited in their ability to automatically detect microarrays and quantify intracellular properties [28]. Proprietary microarray software such as Quantarray (Packard BioChip Technologies, Billerica, MA, USA) and Genepix (Molecular Devices, Sunnyvale, CA, USA) allow users to define arrays individually in one image but are limited in their ability to automatically recognize the bounds of the array [29, 30]. Recently, several algorithms have been developed to individually analyze droplet microfluidic data including screening target cells within droplets, tracking droplet morphology and velocity, or tracking single-cell movement trajectories within droplets [31–33]. While these algorithms can be integrated into experimental platforms to extract superficial droplet or cellular information, they are often restrained to a single analysis metric and are limited in their ability to quantify cell encapsulation information and intracellular properties.

The goal of this work was to develop an algorithm called FluoroCellTrack that could rapidly analyze a wide range of fluorescent microscopy images from different droplet microfluidic applications. The algorithm was developed using Python 3.6.0 (Python Imaging Library, Python Software Foundation), considering the advantages of it over other programming languages such as MATLAB, C++ and Java [34]. The algorithm was designed to handle overlay images obtained using fluorescent microscopy which consisted of both brightfield and fluorescence overlays. To demonstrate the wide-range utility of FluoroCellTrack, it was utilized for three different applications: (1) to characterize and quantify a heterogeneous population of single OPM2 cancer cells based on their dynamic response to different concentrations of a drug; (2) to distinguish cells from clusters of spectrally independent luminescent nanoparticles (NPs) co-encapsulated in droplets which were successfully used as droplet trackers in prior work by Vaithiyanathan et al. [35] and (3) to quantify heterogeneous uptake of fluorescent cell penetrating peptides (CPPs) in single cancer cells through intracellular fluorescence as described in previous work by Safa et al. [36]. The algorithm was able to automatically (i) distinguish droplets from cells, (ii) distinguish and count live, dead and overlapping cells in each droplet, (iii) retrieve cell and NP co-encapsulation information to track single cells in each droplet, and (iv) quantify the intracellular fluorescence of intact cells. A significant limitation of single cell analysis was the time-consuming nature of manual quantification; however, FluoroCellTrack takes <1 min to count cells and <30 min to quantify intracellular fluorescence, compared to ~60 min and ~20 h using manual analysis. The accuracy of FluoroCellTrack was compared with manual counting of cells and found an average of ~92–99% similarity out of a total population of an average of 320 cells (per experiment) coupled with a 19-fold increase in analysis speed over manual counting. While in this work, FluoroCellTrack was tested on a low-throughput data for the ease of comparison with manual analysis, it can quantify several fluorescence-based, high-throughput and multiplexed data sets obtained from droplet microfluidic platforms in addition to microfluidic cell trap arrays or microdroplet arrays [37, 38]. Beyond microfluidics, this algorithm can also be easily employed to quantify fluorescent cells in platforms such as microarrays, microchannels, 3D micro-environment, microtiter plates, and petri dishes [39, 40].

## Materials and methods

### Cell culture and reagents

The details about cell culture, reagents and equipment used in all the three applications [35, 36, 41] are described in S1 Method.

### Design and fabrication of the microfluidic device

The microfluidic device used here is a two-layered droplet generator which was designed and fabricated as described by Vaithiyanathan et al. [35] and Safa et al. [36]. A brief description on this is presented in S2 Method.

### Fluorescent microscopy: Camera and filter sets

For experimentation, the microfluidic device was mounted on the stage of a fluorescent DMi8 inverted microscope (Leica Microsystems, Wetzlar, Germany) to visualize droplet generation, trapping and to acquire microscopy data. This DMi8 inverted microscope, equipped with a range of different magnifications (5x to 20x), was connected to an ORCA-Flash 4.0 V2 Digital CMOS Camera C11440-22CU (Hamamatsu Photonics K. K., Shizuoka, Japan) through a USB 3.0 interface for image collection. The CMOS camera had a 4.0-megapixel resolution (2048 pixels x 2048 pixels) with a high-speed readout (100 frames/s), while achieving a 1.0 electron (median) readout noise performance. The digital camera provided a very high sensitivity through its micro lens (37,000:1 dynamic range) with the CMOS image sensor providing 6.5 μm x 6.5 μm pixel sizes, thus making this camera the most suitable for dynamic brightfield and fluorescent imaging across a wide range of spectra. The microscope was equipped with external trigger functions and time output functions to control the essential timing control during imaging. The following excitation/emission filters (Chroma Tech. Corp., Bellow Falls, VT, USA) were used: fluorescein isothiocyanate FITC (λ_ex_: 440–520 nm and λ_em_: 497–557 nm); rhodamine (λ_ex_: 536–556 nm and λ_em_: 545–625 nm); filter set 1 (λ_ex_: 370–420 nm and λ_em_: 605–645 nm); and filter set 2 (λ_ex_: 325–355 nm and λ_em_: 505–565 nm).

### Experimental set up

The experimental steps describing sample preparation and execution of the three test systems (dose response analysis of OPM2 cells to Bortezomib using a droplet trapping array, tracking single cells using luminescent nanoparticles in a droplet trapping array [35], and understanding CPP uptake in intact cancer cells by measuring intracellular fluorescence) [36] are explained in S3 Method.

### Quantification of data: Automated image analysis and manual analysis

The FluoroCellTrack algorithm was developed in Python 3.6.0 software language (Python Software Foundation, Wilmington, DE, USA) using OpenCV (Open Source Computer Vision, Intel Corp.). Despite being slower than C/C++, Python was preferred in this work due to its ease and simplicity: the syntax in Python involves fewer steps as compared to C++ or Java. Another advantage of Python exploited here was its use alongside OpenCV, an open source machine learning software library that consists of a wide range of programming functions for real-time computer vision applications in pattern recognition, event detection, and artificial intelligence. OpenCV has its application programming interface (API) with several other programming languages and the interface in Python is called the OpenCV-Python which combines the best features of OpenCV-C++ API and Python. This feature allowed for writing computationally intensive codes in C/C++ and using a Python wrapper to use these codes as Python modules. This served as a two-in-one advantage. First, it allowed the code to run as fast as the C/C++ script and second, it gave the programmable ease of Python. OpenCV was used instead of the conservative Pillow (Python Imaging Library) due to the strong focus and better computational efficiency in wide range of applications.

In FluoroCellTrack, highly optimized libraries of OpenCV-Python (e.g., NumPy, SciPy, scikit and matplotlib) were used to execute several numerical operations, thus making this an appropriate tool for fast prototyping of computer vision problems. FluoroCellTrack was tested for its performance on various experimental test systems using a 64-bit Operating System with a processor of base and turbo speeds of 2.50 GHz and 2.70 GHz. All collected microscopy data consisted of images with overlaid brightfield and fluorescent channels, which were processed and analyzed, resulting in the extraction of the required information using a series of 16-bit processing and operational steps. For multiple types of input, the algorithm was designed to handle folders of microscopy images of all formats (e.g., .*tiff*, .*png*, .*gif*, .*jpeg*, *and* .*bmp*). The acquired results were exported in suitable formats (.*csv or* .*txt*) for further data analysis. The efficiency of this algorithm was calculated by comparing the results obtained from automated quantification to manual quantification. The detailed description of the theory and validation of the automated quantification is given in the following sections. The manual analysis of the first two test systems consisted of counting live and dead single/multiple cells and luminescent NPs within droplets to register for encapsulation data, cell viability data and droplet tracking data [35]. For the third test system, the mean intracellular fluorescence intensities of cells were manually quantified through gray values by drawing a polygon scan region of interest (ROI) across each cell using LAS X software 3.3.0. This fluorescence intensity signal was normalized against the background noise obtained from each experiment thus giving a range of normalized intensity values which were further used for statistical and data analyses as described by Safa et al. [36].

## Theory

**Fig 1:**
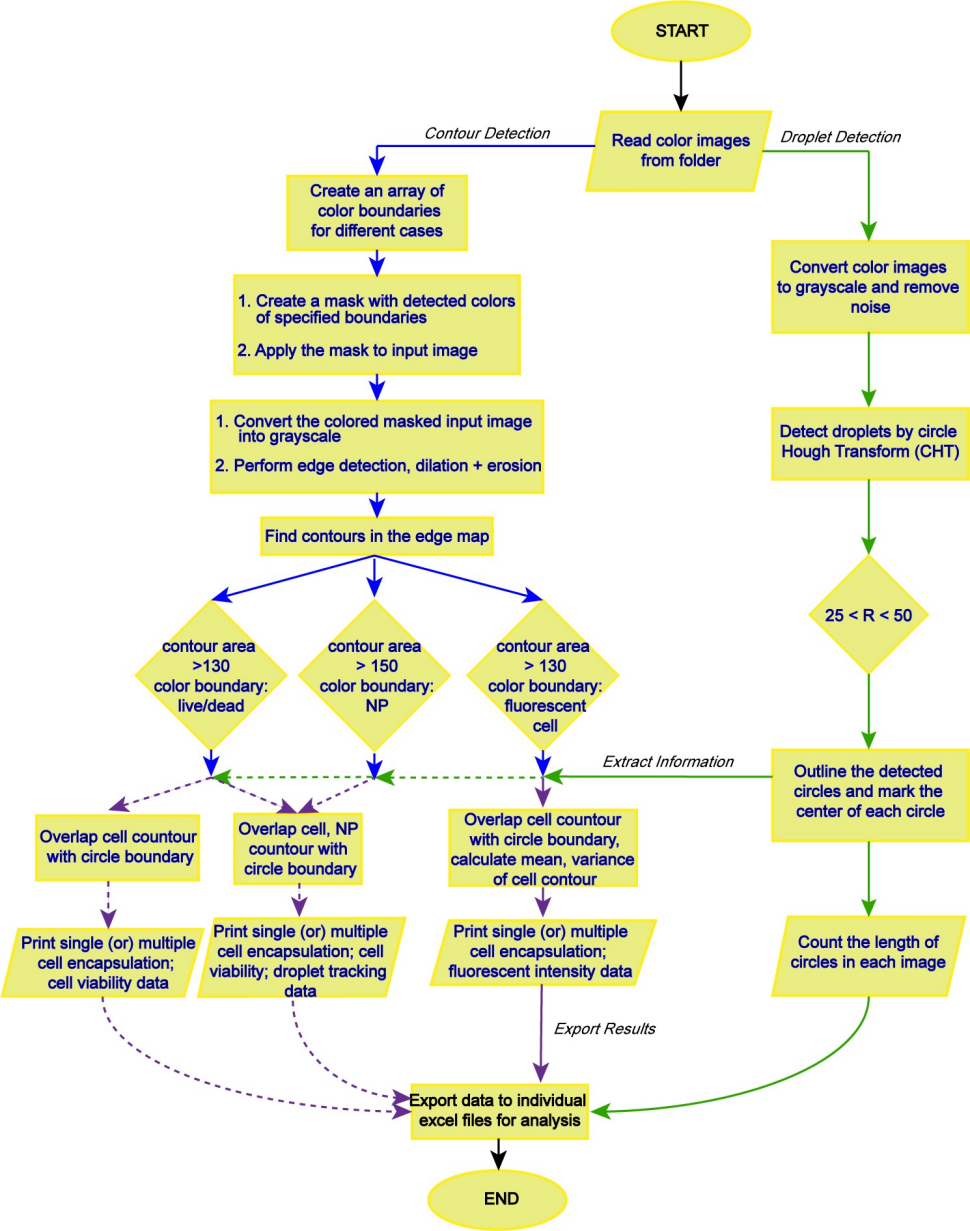
Outline of the FluoroCellTrack algorithm. The starting and ending points are depicted by ovals, the input and output commands are depicted by parallelograms, the processing steps are rectangles, and the decision steps are diamonds. The green arrows denote the steps in droplet detection, the blue arrows show contour detection, and the purple arrows represent information extracted from combining droplet and contour outputs. The dotted lines represent extended applications of the algorithm.

The FluoroCellTrack algorithm consists of several steps as depicted in Fig 1. This schematic outlines the pipeline that can quantify a wide range of data generated using the microfluidic droplet trapping array. The algorithm begins with a basic feature to read fluorescent microscopic images that were collected and stored in folders. Approximately 30–40 unprocessed, overlaid brightfield and fluorescent images were batch fed into the algorithm. The major steps of the algorithm included: (1) pre-processing to remove noise from the images; (2) feature detection including droplet and contour detection; (3) post-processing to extract essential information from the images; and (4) exporting the results for additional analyses. All the processing steps involved 16-bit processing techniques.

### Droplet detection

The steps used to identify droplets trapped in the microfluidic device were accomplished using Hough Transform, which is a basic technique for digital image processing to detect different shapes.[42] Here, a specific Circular Hough Transform (CHT) was used to extract information about circles from the input images [43, 44]. Prior to detecting the circles (droplets), the input microscopy overlay image (*img*) was smoothened to remove noise over signal as shown in Eq (1), where *S* is the smoothening function and the extent of smoothening is decided with a median filter, *M*.

$${img}_{\left(D,gray\right)}=S*(img,M)$$ (1)

The smoothened grayscale image (*img*_*(D*, *gray)*_*)* was further used as an input for the CHT function to detect circles. The parameterization of a circle was described with three features: the center *(a*,*b)* and radius *R*. The equations of a circle in *xy* space is written as:

x=a+Rcosθ (2)

y=b+Rsinθ,0<Ѳ<360 (3)

Using CHT, triplets *(x*,*y*,*R)* were identified that were highly likely to be the parameters of the circle. Next, it was assumed that R was a known quantity and *(a*,*b)* were unknown due to the fact that droplets with known radii must be detected from the input image. Eqs (2) and (3) were rearranged into *ab* space as follows:

$$a=x_{n}-Rcos\theta$$ (4)

$$b=y_{n}-Rsin\theta ,0<Ѳ<360$$ (5)

As θ sweeps between 0° - 360°, every point *(x*_*n*_,*y*_*n*_*)* in the *xy* space is equivalent to a circle in the *ab* space. The steps used to detect circles with respective parameterization are as follows:

1. Load the smoothened image. The smoothened grayscale image *img*_*(D*,*gray)*_ was considered as the *input array*.
2. The known R values of the droplets were given minimum and maximum range of 25 and 50 microns (due to occasional experimental inconsistency leading in generation of droplets of varied sizes). For these known *R* values, detect edges in the *xy* space and create a binary image.
3. For every detected ‘edge pixel’ in the *xy* space, an equivalent circle is drawn in the ab space. This edge detection step involves efficient thresholding.
  $$D_{centers}=CHT({img}_{\left(D,gray\right)},param)$$ (6)
  where, $f(CHT)=A\sum_{i=0}^{n}\nabla g(x_{i},y_{i})[\begin{array}{ccc} x_{i},y_{i} & \cdots & x_{i},y_{n}\\ \vdots & \ddots & \vdots \\ x_{n},y_{i} & \cdots & x_{n},y_{n} \end{array}]=[a_{max},b_{max}]=D_{centers}$
  $$N_{droplets}=len(D_{centers})$$ (7)
  $$C_{mask}=f_{CLR}(\sum_{i=0}^{n}{img}_{\left(x_{i},y_{i}\right)},BGR_{\left[upper,lower\right]})$$ (8)
  $$C_{gray}=C_{mask}\;⃘img$$ (9)
  $$C_{edge}=E(C_{gray},T)$$ (10)
  where, $E=\left\{ \begin{array}{c} C_{smooth}=S*(C_{gray},M)\\ C_{edge}=M_{T}(x,y).(\nabla f(x_{i},y_{i})[C_{smooth}])\;for\;T_{min}<M(x,y)<T_{max} \end{array}\right.$
  $$C_{dilate}=K⊕C_{edge}$$ (11)
  $$C_{erode}=E⊖C_{dilate}$$ (12)
  $$C_{out}=P[{\left(x,y\right)}_{C_{erode}}∊I]$$ (13)
  where, *I* is the predefined label
4. Cast ‘votes’ in the accumulator for every point in the ab space. The accumulator is a 2D array which holds the values of two parameters *a*,*b*.
5. Points with the highest votes in the *ab* space were detected as droplet centers, which are stored in the *output array*, *D*_*centers*_.

The above steps describing the droplet detection are performed mathematically as shown in Eq (6), where CHT is the mathematical function performed on the smoothened input image (*img*_*(D*,*gray)*_*)* with parameterization *(param)*, to yield the output array, *D*_*centers*_. The CHT function as described earlier involves the detection of droplet centers *[a*_*max*_, *b*_*max*_*]* by casting votes in the accumulator *A* when the hough gradient, *∇g* scans across all the pixels (*x*_*i*_,*y*_*i*_ to *x*_*n*_,*y*_*n*_) of the *img*_*(D*,*gray)*_. Once the CHT is implemented to the smoothened input image, the algorithm checks for the detected circles with the parameters as described. If the detected circles satisfy the parameters, the boundaries and centers of these circles are traced and printed. Thus, the number of droplets *N*_*droplets*_ is obtained by the length of the output array, *D*_*centers*_ as shown in (7).

### Contour detection: Fluorescent regions of interest

The fluorescent cells (NPs or any fluorescent regions) encapsulated within droplets in the microfluidic device were considered as contours. This detection step included several defining and processing steps outlined below:

1. Define arrays of color boundaries. Specific color boundaries for cells and luminescent NPs were assigned in this step.
2. Create a mask by detecting colors with specified boundaries.
3. Load input image, apply the mask, convert it to grayscale, and smoothen it.
4. Perform edge detection, dilation, and erosion. This is followed by segmentation to detect attached contours.
5. Find contours.

Arrays of specific color boundaries were created to detect contours of specific interest. The contours of interest for cells were the colors green, red, and yellow. Yellow was used to denote the overlap between the live (green) cells and dead (red) cells in the small population that showed both fluorescent signals. The fine elements of image processing and the availability of the broad color palette was exploited here where color codes of magenta and blue were used for Europium (Eu^3+^)-doped NPs and Terbium (Tb^3+^)-doped NPs to avoid color overlap with live (also GFP) and dead cells (also RFP). The arrays for each color had a lower and an upper boundary limit to detect color in terms of blue, green, and red for BGR vector values. Once the values of lower and upper boundaries were defined, the colors with specified boundaries were detected from the input image (*img*) using respective *BGR*_*[upper*, *lower]*_. After this step, a mask, *C*_*mask*_ was created by detecting respective colors when the function *f*_*CLR*_ was allowed to scan over each pixel *x*_*i*_,*y*_*i*_ of the input image *img*. This mask was then overlaid on the input image which was then converted to grayscale and was smoothened (S) to filter signal from noise, thus yielding *C*_*gray*_ as shown in Eqs (8) and (9). This masking step was done to ensure the exact mapping of fluorescent layer with that of the brightfield, where even the weakest signal from the contour will be finely detected.

The masked, image (*C*_*gray*_) was subjected to several processing steps before detecting contours. The first processing step was to detect edges using Canny Edge Detector [45]. As described in Eq (10), the Canny Edge Detector involved smoothening of *C*_*gray*_ with median filter *M*, followed by computing the gradient, *∇f* of this smoothened image to finally threshold it through non-maxima suppression (*M*_*T*_) to detect edges. The detected edges between the minimum and maximum threshold values (*T*) were finally used to plot the resultant edge map (*C*_*edge*_). This binary edge map was then subjected to the morphological operations erosion and dilation as shown in Eqs (11) and (12). These two functions were used to process images based on their shapes by convolving structural kernels (D, E) with the edge map (*C*_*edge*_). The dilation function was used to join broken parts of the object edges and accentuate the features. This dilation step was followed by the erosion step, to remove white noise and to detach two connecting objects.

The resultant processed image (*C*_*erode*_) was then put through the random walker segmentation algorithm [46] to distinguish attached contours. As denoted in Eq (13), this segmentation function was executed by comparing each pixel of the image, *C*_*erode*_ to a predefined label (*I*) and thus determining the probability of this comparison to finally enhance local maxima of each of the attached individual contour. The final step was to highlight contours through contour retrieval and approximation method. Each detected, segmented contour was stored as a vector of points in the output array *C*_*out*_. The detected contour values in this array were checked for convexity defects using convex hull adjustment function which was similar to the contour approximation method but, checked for bulged-out or flat curves within a contour and approximated them by drawing a defined boundary. In order to effectively detect live/dead/overlapping (dying) cells and NPs, the outlined cell contours were checked for their area. This step was included to exclude cellular or experimental debris from quantification. In case of measuring intracellular fluorescence corresponding to CPP uptake in intact cells, the mean and variance values of each of the positively detected cell contour boundaries were calculated. Next, information about single versus multiple cell encapsulation efficiencies and droplet tracking data was extracted by convolving the detected droplet center map (*D*_*centers*_) over the detected contours center map (*C*_*centers*_). The droplet centers were used as an axis center and the minimum and maximum R values of 25 and 50 microns were used to scan the overlapped contour positions. The positively overlapped cell counters within droplets gave information about single cell encapsulation versus multiple cell encapsulation in the droplets. This analysis further provided insight on the spatial location of single cells and NPs co-encapsulated in a single droplet to provide information on cellular tracking within droplets. All of these output data were exported to suitable formats (.*csv* or .*txt*) for further population analysis. Additionally, it is to be noted that the trap centers in the droplet microfluidic device follow a hexagonal pattern with an exact radius of 35 μm and are placed equidistantly at 360 μm from each other. This was used as an advantage to eliminate multiple droplets within a trap during image pixel scan, where droplet centers once overlapped with contour centers are tested for equidistance (350-370-pixel length) thus aiding in elimination of multiple droplets.

## Results and discussion

### Droplet and contour detection: Validation of theory

**Fig 2:**
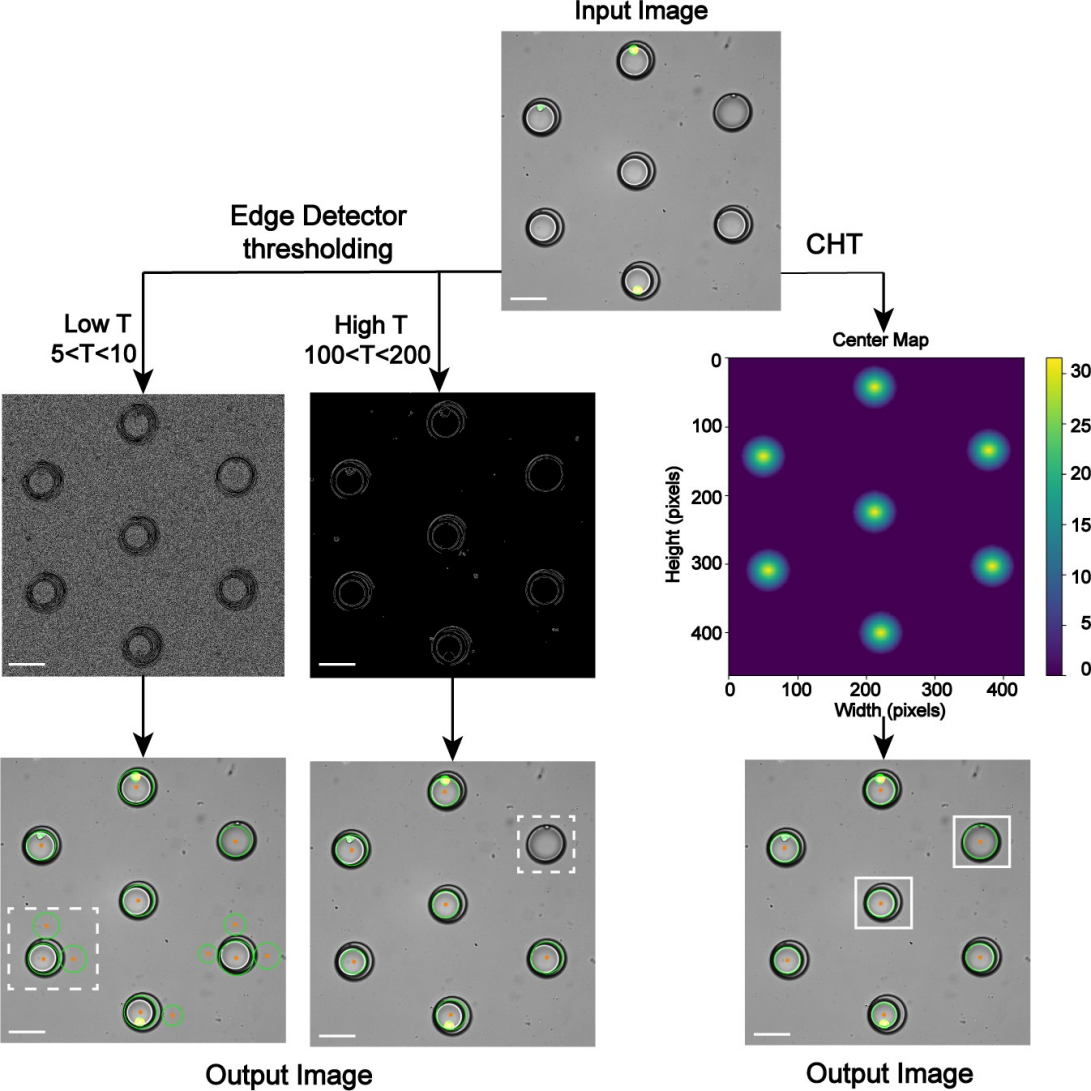
Comparison of edge detector and CHT in detecting droplets. Left: The edge detector function involved manual thresholding resulted in incorrect detection: low T led to false positive values while high T led to data destruction, as highlighted in dashed white boxes. Right: CHT resulted in precise detection of droplets with exact requirement of 25<R<50 microns, as highlighted in white boxes. CHT outlined the droplets with green boundaries and orange centers which were finally counted for further analysis. Scale bar represents 70 μm.

The droplet and contour detection steps of FluoroCellTrack resulted in the successful detection of features, quantification of single versus multiple cells encapsulated in aqueous droplets trapped in the array as well as identifying the individual fluorescent cellular response to different input conditions. FluoroCellTrack was able to read the input folders, each consisting of ~30–40 microscopy images (.*tiff* format was used here). The CHT mathematical function enabled facile and efficient detection of circular features from the images. Droplets with R values ranging from 25 to 50 microns were detected which demonstrated that results from experimental inconsistency could also be included in quantification. This CHT step proved to be a powerful feature detection tool over the edge detection feature, which has been conventionally used [47, 48]. In order to compare CHT and conventional thresholding, both of the algorithms were run side-by-side (Fig 2). Here, manual thresholding with the edge detector function lacked the sensitivity and specificity needed for accurate detection. Specifically, the lower threshold values led to noise retainment resulting in false positives and higher values resulting in data removal (Fig 2, dashed white boxes). Data smoothening with a median kernel followed by CHT led to precise detection of circular features with their centers and boundaries of exactly 25<R<50 microns. Through CHT, the local maxima of each of the droplet was precisely mapped where the detected droplets boundaries and centers were highlighted in green and orange. These marked centers were finally counted to detect the total number of droplets. Since the majority of the droplet microfluidic data consists of circular features of known R values, CHT plays the ideal function in detecting such features. It is to be noted that, the CHT function lags behind in analyzing systems with circular features of unknown R values and thus, it can be replaced by the modified canny edge detector function as recently discussed [49]. Additionally, for systems which generate non-circular droplets (i.e. oval or ellipse), the Fast Ellipse Hough Transform (FEHT), as previously discussed by Xie et al. and Wong et al. [50, 51] can be implemented over CHT. The FEHT works in similar way to that of the CHT with a distinguished parameterization of major and minor axes (circles are nothing but special case of an ellipse, where the major and minor axes are equal). In summary, this droplet detection can be used as a stand-alone step for different applications: in characterizing droplet parameters [32], to quantify lipid droplets shrinkage [52], to detect optimal concentration of fluorescent dye within droplet etc. [53, 54].

**Fig 3:**
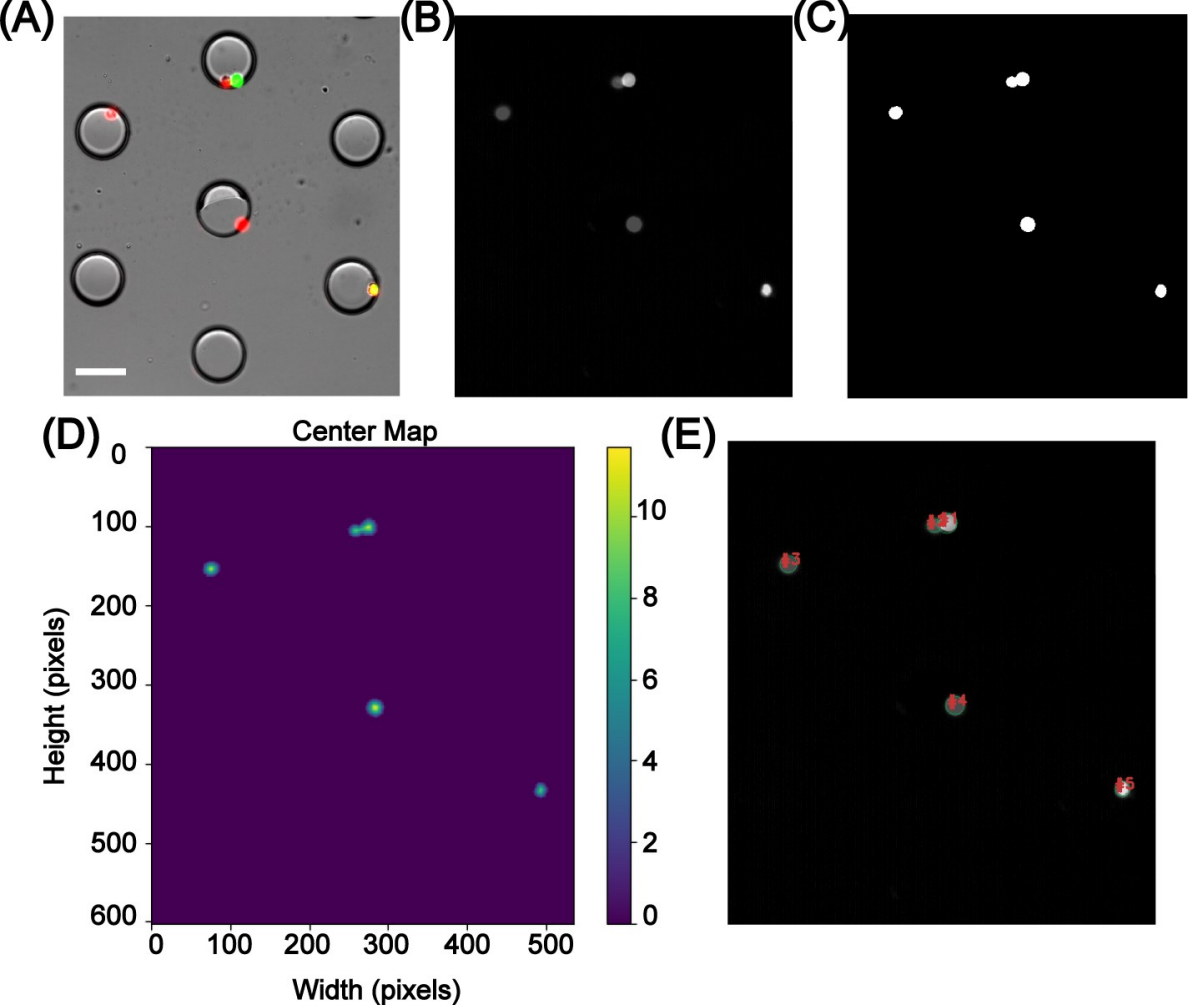
Steps involved in contour detection. A mask was generated from the input image (A) through definition of color boundaries. This mask was convolved with the input image, smoothened and converted to grayscale as shown in (B). Edge detection, dilation and erosion was performed on (B) to yield (C) which was then subjected to random walker segmentation to map the contour centers as shown in (D). The detected contours were finally checked for area where cellular area equal to or greater than 130 pixels were outlined and labeled (E). Scale bar represents 70 μm.

**Fig 4:**
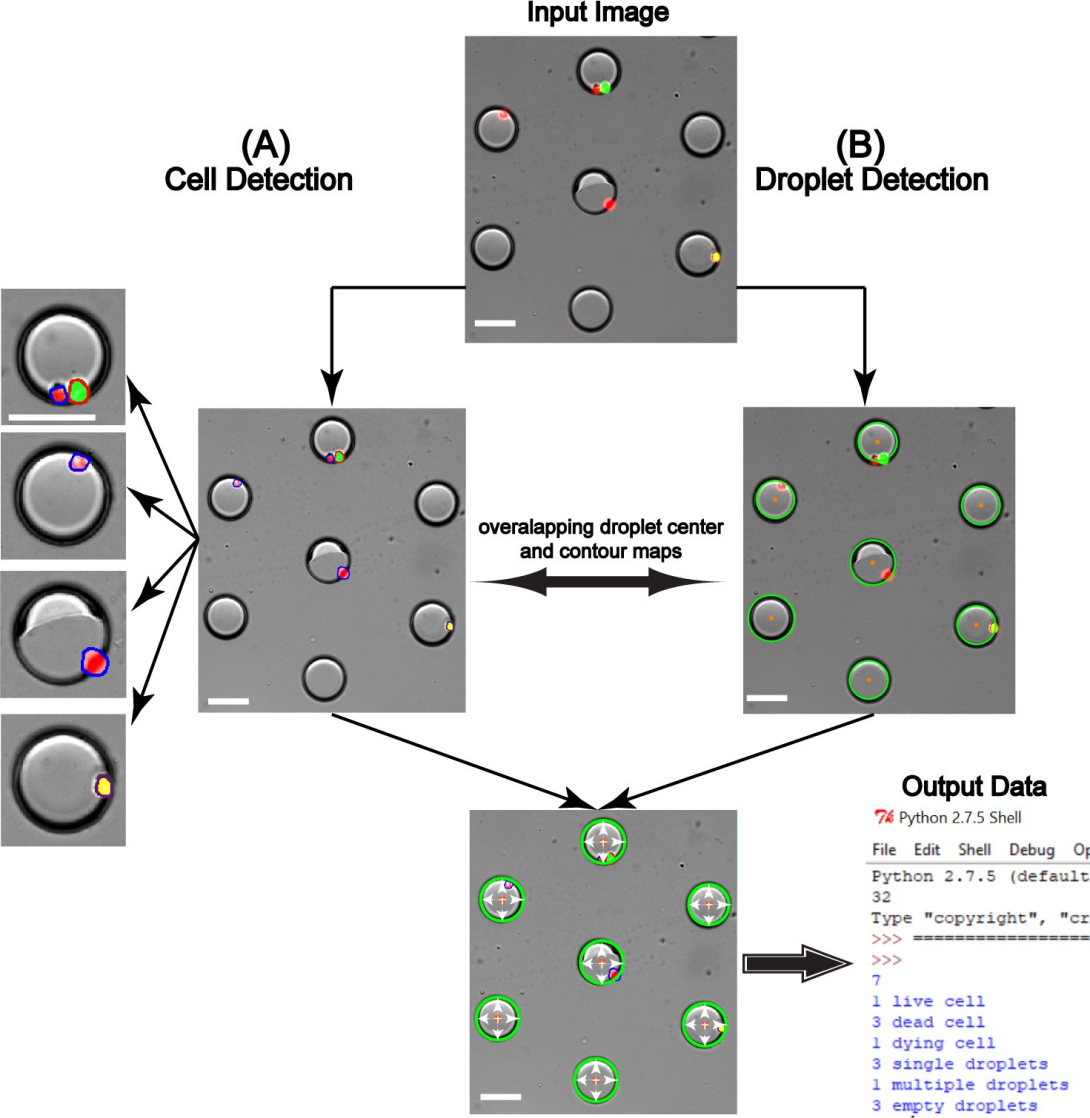
Quantification of encapsulation information. (A) The process of cell detection involved setting of color boundaries, edge detection, segmentation to finally generate ROIs around cells including live cells (green), dead cells (red) and overlapping cells (yellow) outlined in red, blue and purple. Differently colored ROIs are counted for respective type of cells (as shown in output). (B) Detection of droplets through CHT mark them with green outlines and orange centers. The overlapping droplet center map with cell center map and defining droplet axis centers and radius boundaries resulted in generation of encapsulation information in output data. Scale bar represents 70 μm.

Next, contour detection was implemented to detect cellular features as shown in Fig 3. First, a mask was generated by detecting differently colored cells from the input image, Fig 3A. This mask (Fig 3B) was overlapped with the input image and was subjected to edge detection, dilation and erosion to yield Fig 3C, which was then segmented through random walker algorithm to detect and distinguish attached cells. Random walker segmentation algorithm was used over traditional watershed algorithm due to its highly precise probabilistic interpretation of local maxima [55, 56] (Fig 3D). The distinguished contours from this image were then filtered for area, where cellular area equal to or greater than 130 pixels were outlined and labeled as shown in Fig 3E. 130 was selected as the thresholding value due to the mean cellular area being ~140 pixels. This step was essential to exclude debris to eliminate false positives. However, in case of co-culture platforms, where primary cells are of the same size as that of the secondary cells [57, 58], or in systems where the debris is of the same size as that of the cells, specific cellular or organelle staining can be used to distinguish these features and the extensive palette used in computer vision aids in easy detection of such features. This contour detection step used here was found to be technically stronger than the template matching feature during the initial stages of the development of FluoroCellTrack, which is a commonly used technique in digital image analysis to find small parts of an image that match the template image [27]. For instance, similar features with slight deviations in shape, size and orientation were easily detected by the droplet/contour detection steps of FluoroCellTrack but would need a repository of many templates while using the template matching feature, showing that the latter can be highly uneconomical. The broad palette of colors in computer vision can be exploited here to detect any fluorescent cell and as an example here, the contour detection step was implemented to quantify actual cellular viability data as shown in Fig 4. Lower [B, G, R]: [0, 51, 0] and upper [B, G, R]: [153, 255, 153] boundaries were given for live cells and, dead cells were identified with the lower and upper color boundaries at [0, 0, 51]; [153, 153, 255] while overlapping cells were identified with lower and upper color boundaries of [0, 51, 51]; [153, 255, 255]. Cells with overlapping signals in both the green and red filter sets represent cells with compromised plasma membranes (indicative of a dead cell), but with remaining esterase activity to react with the Calcein AM. These cells were noted as ‘dying’ cells to distinguish them from the clean live and dead signals using the other contour boundaries. The contours with specific colors were detected from the input images, where the positively detected live, dead and overlapping cells with an area equal to or greater than 130 pixels were outlined with distinct colors: live cells in red, dead cells in blue and overlapping cells in purple (Fig 4). The outlined cell-center map was overlaid with the droplet center map and scanned to fit within 25<R<50-micron values (denoted by white arrows), to account for single/multiple cell encapsulation information as shown in the output data of Fig 4. This step was essential to calculate the single-cell encapsulation efficiency and to eliminate cells that were freely floating in the device, thus including cells that were only inside the droplets. Additionally, the user can easily eliminate the droplet detection step to use this contour detection as a stand-alone platform (similar to ImageJ and Cell Profiler) to detect and quantify properties of fluorescent cells from other microfluidic or non-microfluidic applications [59–61]. The advantage of FluoroCellTrack over current software tools like ImageJ and Cell Profiler lies in the fact that the former can be employed in non-droplet microfluidic platforms in addition to droplet microfluidics, but the latter cannot be used to quantify high-throughput droplet microfluidic data.

### Quantification of the single cell response to different doses of a drug

**Fig 5:**
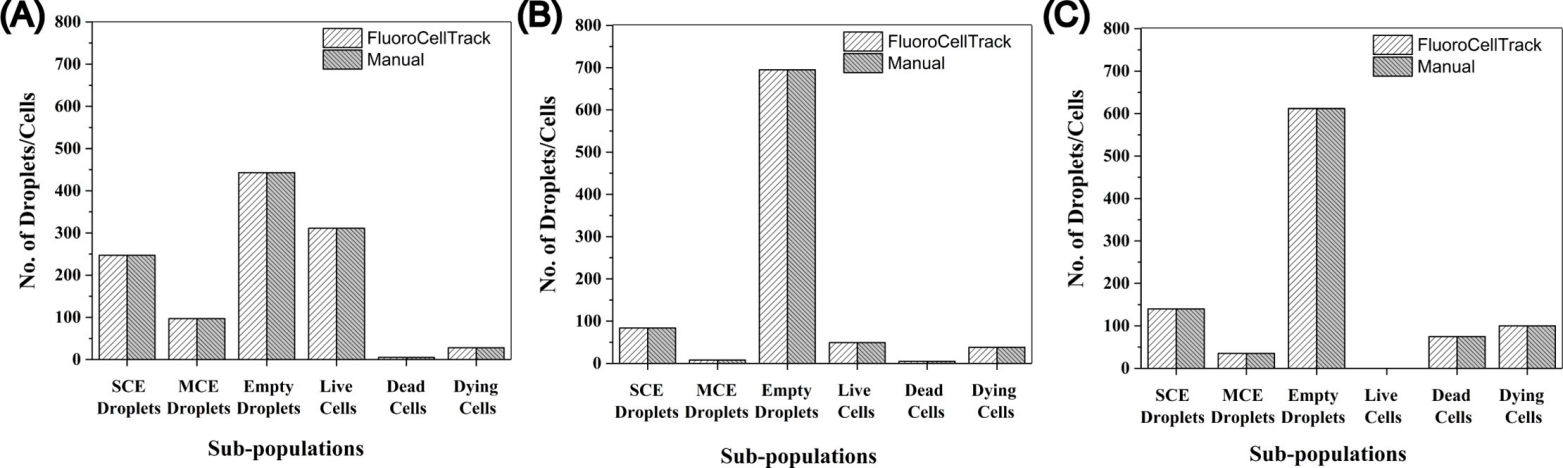
Comparison of FluoroCellTrack to manual inspection to quantify single OPM-2 cell viability. Cells were treated with different dozes of BTZ for 24 h (A), 48 h (B), and 72 h (C). An average of ~99% similarity was observed between the outputs of FluoroCellTrack and manual inspection in terms of single cell and multiple cell encapsulation (SCE and MCE) and assessment of cell viability. A minimum of 275 cells and n = 787 droplets were analyzed in each case.

An important application of any automated platform is to count cells and sort them based on their fluorescent response. As an example, the dose-dependent single cell response of OPM-2 cells to a clinical inhibitor, Bortezomib was observed. FluoroCellTrack quantified the heterogeneous responses of single OPM-2 cells treated with three doses of Bortezomib (1 nM, 10 nM and 100 nM) for three incubation periods (24 h, 48 h, and 72 h). A minimum of 275 cells (average 320 cells) and 787 droplets were analyzed for each condition at each time point and as a result, the cells were found to exhibit a decrease in viability at increasing concentrations and incubation times (S1 Table). To determine the accuracy of the algorithm, the results from the automated analysis were compared to a manual inspection of the images (Fig 5). The results obtained by FluoroCellTrack demonstrated an average of ~99% similarity for a triplicate analysis of the three described conditions, while requiring only <1 min to quantify the entire population of cells compared to the ~60 min of for manual analysis. Beyond sorting cells based on cellular viability, this algorithm can easily be applied to several different platforms where fluorescent proteins, quantum dots, biosensors induced dynamic responses of cells are used as a metric to detect intracellular signaling, protein-protein interaction etc. [14, 62–64].

### Tracking of co-encapsulated single cells and luminescent nanoparticles

**Fig 6:**
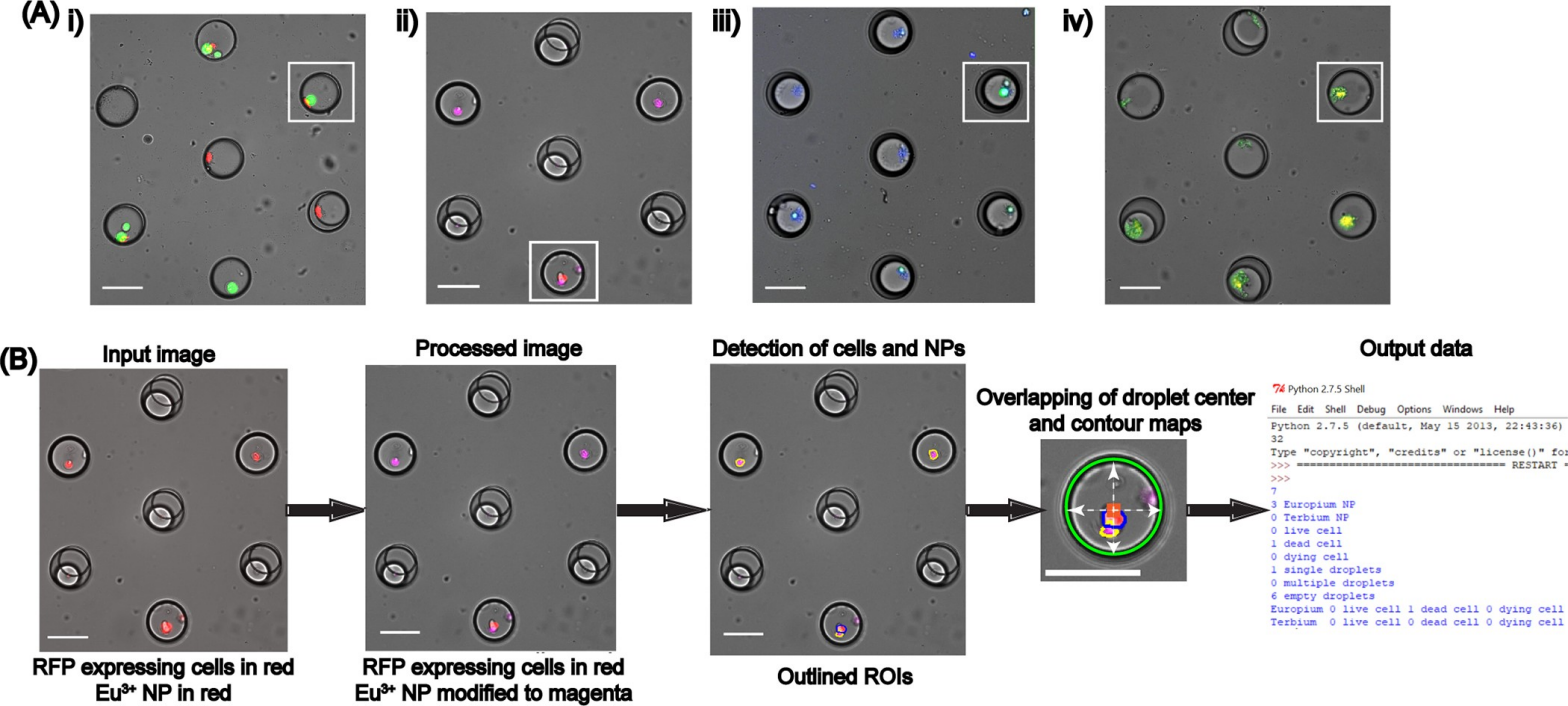
Identification of co-encapsulated single cells and NPs. A) Representative images of (i) GFP-HeLa cells and (ii) RFP-MDA MB 231 cells tracked by Eu^3+^-doped NPs and (iii) GFP-HeLa cells and (iv) RFP-MDA-MB-231 cells tracked by Tb^3+^-doped NPs. White boxes denote droplets co-encapsulated with cells and NPs. Eu^3+^-doped NPs and Tb^3+^-doped NPs are colored magenta and blue in (ii) and (iii) to distinguish them from RFP- and GFP-expressing cells. B) Approach to quantify droplet tracking data using an image from RFP-MDA-MB-231 cells co-encapsulated with Eu^3+^-doped NPs. Eu^3+^-doped NPs are outlined in yellow while RFP-MDA-MB-231 cells are outlined in blue. The single droplet represents the RFP-MDA-MB-231 cell being tracked by Eu^3+^-doped NPs. Scale bar represents 70 μm.

The advantage of fluorescence microscopy coupled with automated analysis by FluoroCellTrack was exploited to quantify cellular tracking data in droplets that co-encapsulated single cells with luminescent nanoparticles as shown in Fig 6. The image acquisition system was designed with four different filter sets to capture GFP/live cells, RFP/dead cells, Eu^3+^-doped NPs, and Tb^3+^ doped- NPs without any overlap between the images (Fig 6A). A similar approach for droplet identification and contour detection was applied here as described above. Luminescent nanoparticles were distinguished from cells based on the definition of color boundaries which was followed by area thresholding. An example of these steps is shown in Fig 6B for RFP-expressing MDA-MB-231 cells co-encapsulated with Eu^3+^-doped NPs. The process of contour detection involved two parallel loops to test for cellular color boundaries and for NP color boundaries. Since Eu^3+^-doped NPs and Tb^3+^-doped NPs had visual outputs in red and green (not to be overlapped with the output colors of dead and live cells), the Eu^3+^-doped NPs and Tb^3+^-doped NPs were assigned the color boundaries of magenta and blue to prevent misidentification with RFP/dead cells and GFP/live cells (Fig 6B). The color boundaries for cells were the same here as described above while the Eu^3+^-doped NPs were detected at lower and upper boundaries of [51, 0, 51]; [255, 102, 255] and Tb^3+^-doped NPs were detected between [51, 0, 0]; [255, 51, 51]. Following cell segmentation, the positive color-based selected contours were looped through area thresholding. Cell contours and NP contours equal to or greater than 130 and 150 pixels were filtered and any respective contour area below these values were ignored. 150 was selected as the area threshold value for NPs due to the mean area for NP clusters being 170. The detected contours in the specific boundaries of cells and NPs were outlined with distinct colors: Eu^3+^-doped NPs were outlined in yellow and RFP-MDA-MB-231 cells were outlined in blue (Fig 6B) while Tb^3+^-doped NP were outlined in orange and GFP-HeLa cells were outlined in red. The detected individual cell center and NP center maps were overlapped with droplet center maps and scanned for droplet boundaries to obtain information on co-encapsulation and droplet tracking. Following this, the droplet centers were finally checked for equidistance to eliminate multiple droplets as shown in output image of Fig 6B.

**Fig 7:**
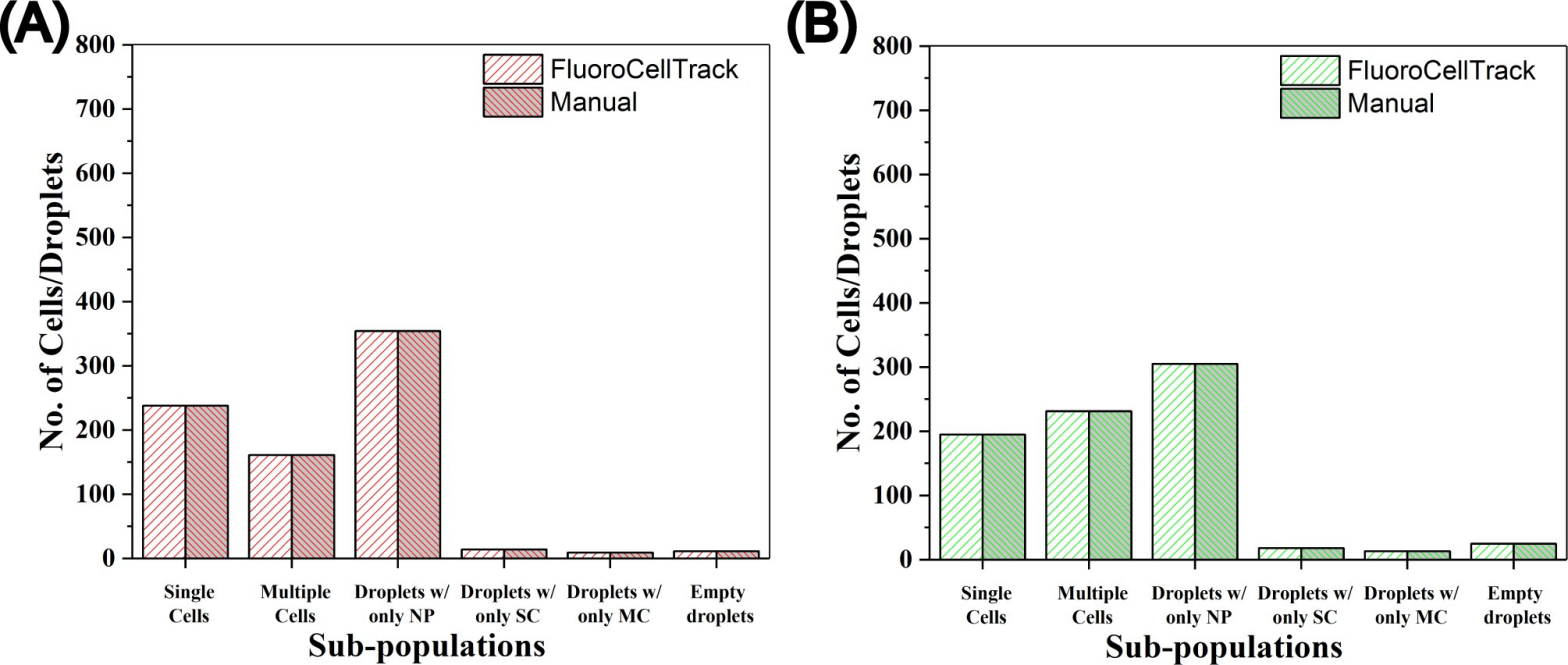
Comparison of FluoroCellTrack to manual inspection of co-encapsulation droplet tracking data. Droplet subpopulation identification was performed for RFP-expressing MDA-MB-231 cells tracked by (A) Eu^3+^-doped NPs and (B) Tb^3+^-doped NPs. SC-single cells, MC-multiple cells. n = 787 droplets were analyzed for each experiment.

FluoroCellTrack was able to identify and sort different subpopulations of droplets with respect to single versus multiple cell encapsulation coupled with droplet tracking by co-encapsulation with rare earth (RE)-doped NPs (S2 Table). The output from FluoroCellTrack was compared to manual assessment of the data (Fig 7), where a maximum of 787 droplets were analyzed per experiment and was found to exhibit an average of ~97–99% similarity to manual counting for a duplicate analysis of each case, while taking <5 min to complete the analysis compared to ~60 min required for manual inspection. This algorithm was further used to quantify other tracking experiments involving live, dead, and overlapping MDA-MB-231 cells with both RE-doped nanoparticles with similar similarities (as discussed by Vaithiyanathan et al. in [35]). The ability to assign different color boundaries to distinguish cells from NPs highlights the utility of FluoroCellTrack in both cellomic and genomic applications. This algorithm can be used as an effective tool to analyze high-throughput multiplexed droplet microfluidic data [37, 65], where more such luminescent NPs or other fluorescent trackers like droplet barcodes could be used to simultaneously track single cells from different input conditions on the same platform or in case of single cell sequencing (DNA or RNA) [37, 66, 67]. While this algorithm successfully quantified tracking information through cellular co-encapsulation, tracking can also be done in this system by simply labelling the droplets. The hexagonal geometry of trap centers in such droplet microfluidic array with each of the traps being equidistant at 360 μm from each other can be overlapped with the detected droplet centers in the process of labelling. Similar parameters can be used in different droplet microfluidic platforms in order to track droplets or other features.

### Quantification of intracellular fluorescence intensity corresponding to cell penetrating peptide uptake in intact single cells

**Fig 8:**
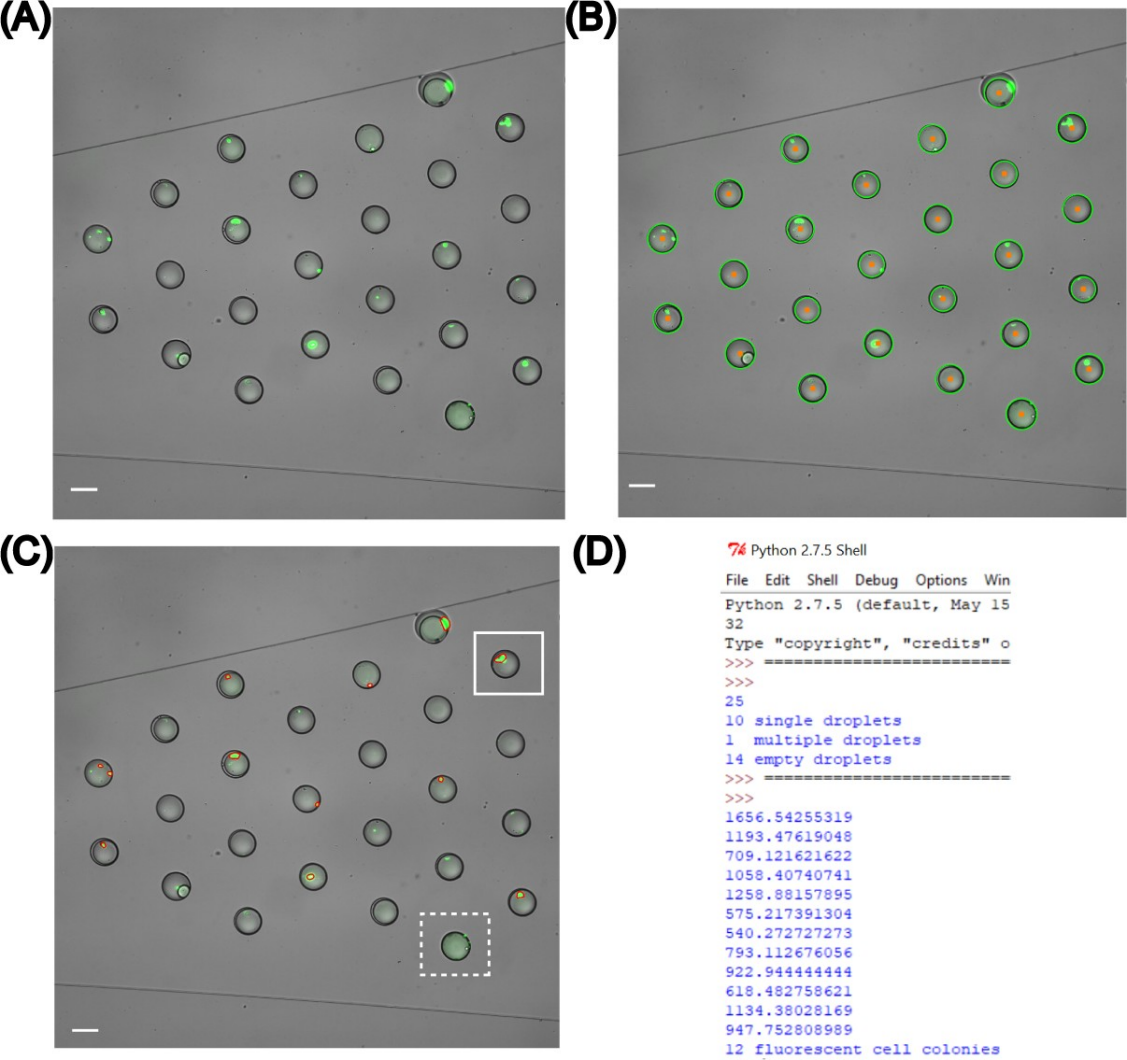
Quantification of intracellular fluorescence using FluoroCellTrack for the uptake of 50 μM ARG. The input image (A) of single HeLa cells containing the ARG peptide was processed to detect droplets (B). (C) Cell detection required area thresholding to filter out debris (dashed white box) from cells (white box) trapped in droplets. (D) Overlapping of droplet center and contour maps produced output data consisting of encapsulation and intracellular fluorescence information.

Prior work by Safa et al. [36] quantified CPP uptake in intact cells using a droplet trapping array; however, the manual quantification of each experiment took ~20 h. FluoroCellTrack was tested to quantify the permeability of two novel CPPs reported by Safa et al. (RWRWR and OWRWR) in addition to two commercially available CPPs (ARG and TAT) [36, 41]. The analysis of intracellular heterogeneity required enhancing the FluoroCellTrack algorithm to be able to distinguish subtle differences in fluorescent intensity of the cells. The algorithm was tuned using data from an experiment quantifying the uptake of 50 μM ARG in single, intact HeLa cells (Fig 8). Peptide uptake in intact cells was visualized using FAM-tagged peptides (Fig 8A). Droplets were detected using the methods described above (Fig 8B). Intact cells were detected between the color boundaries [0, 51, 0] and [153, 255, 153] followed by segmentation to discern between cells and debris. This was accomplished using an area thresholding step to remove anything below 130 pixels (Fig 8C). The contour center map was overlaid with the droplet center map to provide information on the droplet count and cell encapsulation efficiency as shown in the output data from Fig 8D. The mean pixel values within each of the contours were extracted and tabulated to quantify intracellular fluorescence. These values were in the range of [0–65536] pixels and corresponded to the fluorescence uptake of the CPPs within each cell (an example output intracellular fluorescence data was obtained and is shown in Fig 8D). Each of these mean cellular fluorescence intensity values were exported to .*csv* format for further population analysis. Representative data of HeLa cells incubated with 50 μM concentrations of the four CPPs are shown in S3 Table, which list the mean fluorescence values from each experiment. The values calculated by FluoroCellTrack were ~92–98% similar on an average, to those obtained by manual analysis of the population of cells [36]. The small variation between the values calculated by FluoroCellTrack and manual inspection could be attributed to the bias in manual quantification which involved manual boundary identification over automated identification using FluoroCellTrack. A similar trend was observed with lower concentrations of these peptides (10 μM), which was also captured by FluoroCellTrack. The time required to analyze each data set was dramatically reduced using FluoroCellTrack which took <30 minutes/experiment compared to >20 hours/experiment needed for manual inspection.

**Fig 9:**
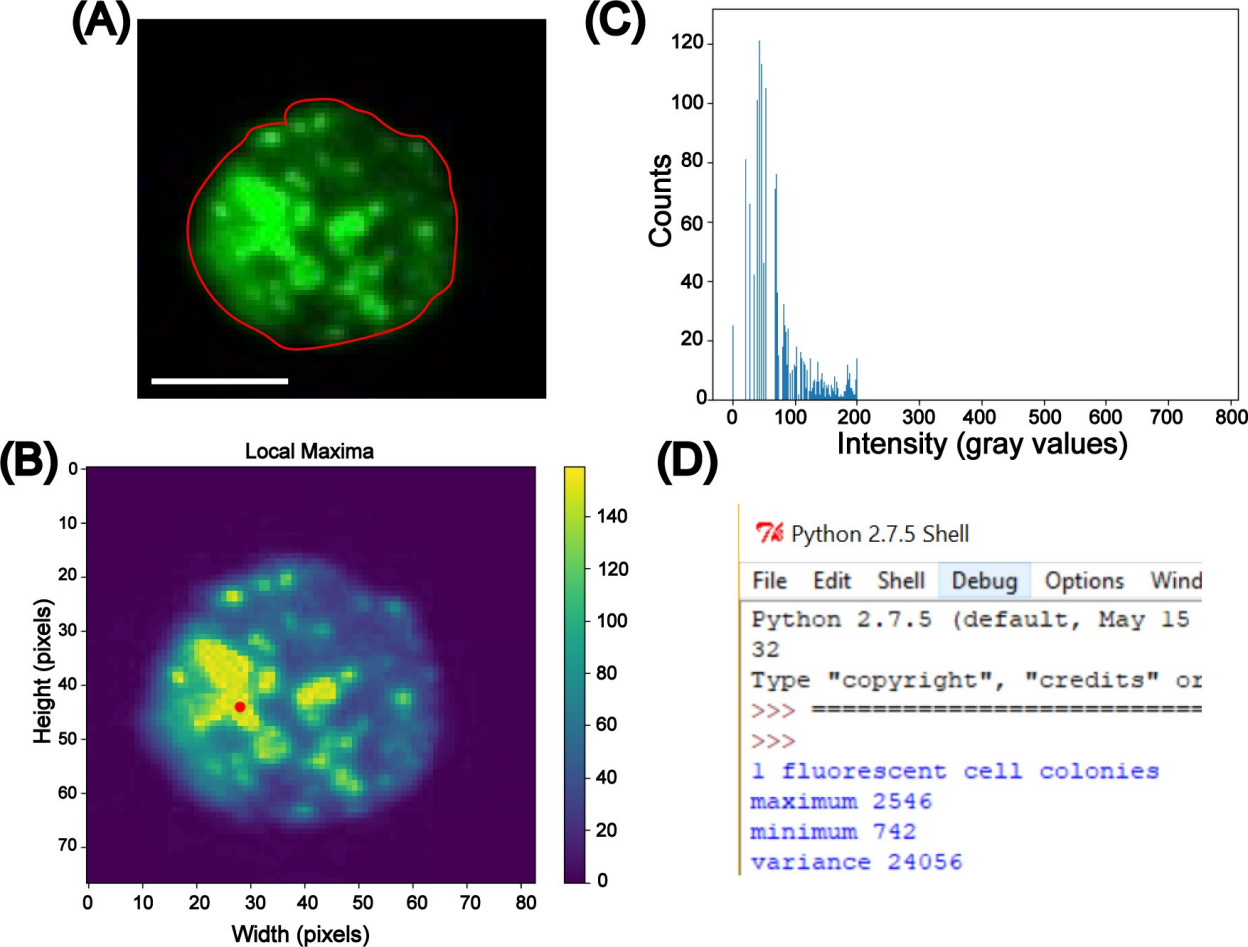
Quantification of intracellular parameters to understand heterogeneous peptide uptake. (A) The outlined fluorescent cell from the fluorescent channel of the input image was traced for its center map and the center was detected in red shown in (B). The 8-bit histogram plot was generated to observe the frequency of grayscale intensity values in (C) leading to the generation of maximum, minimum peak values of intensity along with variance in intensity values which is finally mapped into a 16-bit scale as shown in the output data (D). Scale bar represents 10 μm.

In addition to quantifying mean fluorescence values, FluoroCellTrack was able to record minimum, maximum and variance in pixel values within each cell to further characterize intracellular peptide distribution. Fig 9 contains example values obtained for a single cell incubated with 50 μM OWRWR, for which the maximum, minimum and variance values of grayscale intensity values were obtained from automated quantification (2546, 742, 24056). This was similar in trend to the values obtained by manual quantification (2387, 801, 24013), highlighting the utility of this algorithm for a broad application in understanding population heterogeneity. Beyond this broad application in understanding population heterogeneity through intracellular fluorescence, this algorithm can be combined with Spatial Intensity Distribution Analysis (SpIDA) to understand the cellular distribution of fluorescent stains or markers [68, 69].

## Conclusions

The multistep Python algorithm, FluoroCellTrack was developed to process and analyze folders of microscopy images obtained from global droplet microfluidic experimentation. The algorithm has several distinct features including the automatic detection of droplet subpopulations (e.g., empty droplets, droplets with single cells, droplets with multiple cells), the quantification of single cell responses to drugs, and the quantification of intracellular fluorescence in intact cells. FluoroCellTrack was successfully implemented in three different systems: (i) live/dead subpopulation studies to understand cellular responses to different doses of drugs, (ii) quantification of cell and nanoparticle co-encapsulation for droplet tracking information, and (iii) quantification of CPP uptake in single intact cells based on fluorescent intensity. This algorithm was found to be superior to the commonly used feature detection techniques like Edge Detector thresholding, template matching and had well-defined and precise steps to eliminate false positives such as debris (e.g., cell or peptide) across the trapping array. Manual control analyses conformed with the Python algorithm with an average similarity of ~92–99% from a mean population of 320 cells. Moreover, automated image analysis took about <1 min to count all the cells trapped in the device and <30 min to quantify the fluorescence intensity across the entire population of cells, proving it to be a powerful tool for microscopy data analysis. This was far superior to the ~60 min required for manual cell counting and ~20 h needed for manual analysis of intracellular fluorescence. While the work here demonstrated the utility of FluoroCellTrack with a low-throughput droplet data (~787 droplets), the algorithm has a potential to quantify high-throughput droplet data (~10,000 droplets) in a couple of hours in comparison to days of manual analysis. Finally, FluoroCellTrack was found to overcome the limitations of existing non-droplet microfluidic algorithms and has the potential to be integrated with several different types of microfluidic devices, trapping arrays and non-microfluidic platforms with easy user-defined modifications. Beyond tracking and quantifying intracellular fluorescence, this algorithm has multiple applications in tracking cellular movements, through time-lapse imaging and position detection and can also be potentially extended to understand subcellular molecular processes by analyzing intracellular localization of biochemical stains through intracellular pixel quantification.

## Supporting information

S1 Method Cell culture and reagents. (DOCX)

S2 Method Design and fabrication of the microfluidic device. (DOCX)

S3 Method Experimental set up. (DOCX)

S1 Table Automated quantification of single OPM-2 cell response to different doses of Bortezomib. A minimum of 275 cells and n = 787 droplets were analyzed for each case.
(DOCX)

S2 Table Quantification of droplet tracking data by FluoroCellTrack. Information on droplet subpopulations and cell encapsulation obtained from two individual experiments where RFP-expressing MDA-MB-231 cells were co-encapsulated with Eu^3+^-doped NPs and Tb^3+^-doped NPs. n = 787 droplets were analyzed in each case.
(DOCX)

S3 Table Comparison of mean intracellular fluorescence representative of CPP uptake in HeLa cells using FluoroCellTrack. A minimum of 142 cells (maximum 367 cells) were analyzed in these experiments.
(DOCX)
