# Supplementary material for: FluoroCellTrack: An algorithm for automated analysis of high-throughput droplet microfluidic data
Source: PLoS One. 2019 May 1;14(5):e0215337. doi: 10.1371/journal.pone.0215337 (PMC6493727; doi:10.1371/journal.pone.0215337)
Supplement: S1 Method — (DOCX) [file pone.0215337.s001.docx]

**S1 Method: Cell Culture and Reagents**

HeLa cells and green fluorescent protein (GFP)-expressing HeLa cells were maintained in DMEM Media (Corning Inc., NY, USA) with 10% v/v fetal bovine serum (FBS – VWR Life Sciences Seradigm, Radnor, PA, USA). OPM-2 cells were maintained in RPMI 1640 media (Corning Inc., NY, USA) supplemented with 12% FBS, 21.8 mM glucose, 8.6 mM HEPES -pH 7.4 (Sigma Aldrich, St. Louis, MO, USA) and 1.0 mM sodium pyruvate (Thermo Fisher Scientific, Waltham, MA, USA). MDA-MB-231 cells and red fluorescent protein (RFP)- expressing MDA-MB-231 cells were cultured in DMEM media supplemented with 10% v/v HyClone Cosmic Calf Serum (VWR Life Sciences Seradigm), 1% MEM Essential Amino Acids, 1% MEM Non-Essential Amino Acids (Quality Biological Inc., Gaithersburg, MD, USA), 1 mM sodium pyruvate and 6 µL insulin/500 mL media (Insulin, Human Recombinant dry powder - Sigma Aldrich).

The reagents and equipment used for the synthesis and characterization of luminescent NaYF_4_:RE^3+^ (rare earth, RE =Europium-Eu^3+^, Terbium-Tb^3+^) nanoparticles are explained in the previous work by Vaithiyanathan et al. [1]. Calcein AM (live stain) and ethidium homodimer-1 (dead stain) were purchased from Life Technologies- Thermo Fisher Scientific, Waltham, MA, USA. The proteasome inhibitor Bortezomib (BTZ) and microtubule inhibitor Paclitaxel (PTX) were obtained from VWR Life Sciences Seradigm, and R&D Systems, Inc. Minneapolis, MN, USA. The commercial cell penetrating peptides (CPPs), FAM-tagged TAT and FAM-tagged ARG (FAM denotes the commonly used fluorescent dye 5(6)- carboxyfluorescein) were purchased from AnaSpec, Fremont, CA, USA. Two experimental peptides (RWRWR and OWRWR) were synthesized as described by Safa et al. [2, 3].

References

1. Vaithiyanathan M, Bajgiran KR, Darapaneni P, Safa N, Dorman JA, Melvin AT. Luminescent nanomaterials for droplet tracking in a microfluidic trapping array. Analytical and bioanalytical chemistry. 2019;411(1):157-70.

2. Safa N, Anderson JC, Vaithiyanathan M, Pettigrew JH, Pappas GA, Liu D, et al. CPProtectides: Rapid uptake of well‐folded β‐hairpin peptides with enhanced resistance to intracellular degradation. Peptide Science. 2018:e24092.

3. Safa N, Vaithiyanathan M, Sombolestani S, Charles S, Melvin AT. Population-based analysis of cell-penetrating peptide uptake using a microfluidic droplet trapping array. Analytical and Bioanalytical Chemistry. 2019. doi: 10.1007/s00216-019-01713-5.

.
