# Supplementary material for: FluoroCellTrack: An algorithm for automated analysis of high-throughput droplet microfluidic data
Source: PLoS One. 2019 May 1;14(5):e0215337. doi: 10.1371/journal.pone.0215337 (PMC6493727; doi:10.1371/journal.pone.0215337)
Supplement: S2 Method — (DOCX) [file pone.0215337.s002.docx]

**S2 Method: Design and Fabrication of the microfluidic device**

Briefly, the geometry of the microfluidic device was designed in AutoCAD (2015 version, Autodesk, San Rafael, CA), followed by fabrication methods using soft lithography and PDMS (polydimethylsiloxane) replication. The droplet microfluidic trapping array consisted of two 40 µm layers: the bottom main flow channel and the top layer trapping array (total of 80 µm). The bottom layer consisted of two fluidic inlet ports: one for the oil phase and the second port for the aqueous phase. Flowrates of 230 µL/h and 90 µL/h were maintained for the oil and aqueous phases which converged at the flow focusing junction to generate droplets with vertical fins implemented across the bottom layer to aid trapping. 0.2% fluoro-surfactant (Ran Biotechnologies Inc., Beverly, MA, USA) was used with Novec 7500 oil (3M, Maplewood, MN, USA) for the oil phase to stabilize droplet formation. The top layer consisted of a 787-member grid with individual traps having a 70 µm diameter imprinted 40 microns onto the PDMS above the bottom layer. A two-step soft-lithography was used to fabricate the silicon master: starting with generating the bottom fluidic layer using a negative photoresist polymer, SU-8 2025 (Microchem). The SU-8 was deposited on a clean 4” silicon wafer and baked at 65 °C for 10 min followed by a second bake at 95 °C for 20 min. After cooling down, the wafer was exposed to UV light with 1.2 mW/cm^2^ power intensity for 40 s using an iron oxide/chrome photo mask (Front Range) to create the fluidic channels. The wafer was baked again at 65 °C for 15 min and at 95 °C for 30 min, post UV exposure. These steps were repeated to generate the top trapping layer. The silicon wafer was developed with an SU-8 developer solution (Microchem) to remove the uncrosslinked SU-8 to produce the microfluidic patterns. The wafer was finally hard baked at 150 °C for 30 min to increase wafer durability.

PDMS replicas (Slygard 184, Ellsworth Adhesives) were generated by mixing the base agent in a 10:1 ratio with the curing agent, followed by degassing in a vacuum chamber to create a bubble-free mixture. This PDMS was poured on the silicon master and was cured for at least 6 h at 65 °C. Once cured, the PDMS was removed from the wafer, and the inlet and outlet ports were punched using a blunted 18-gauge needle (VWR International, Radnor, PA, USA). The PDMS replicas were permanently bonded to 25X75 mm glass slides (Corning) using an O_2_ Harrick Plasma PDC-32G basic plasma cleaner (Harrick Plasma, Ithaca, NY, USA) with a 30 s exposure to plasma. The devices were left overnight to ensure proper bonding between the PDMS and the glass. The fluidic channels in the microfluidic device were made hydrophobic by Aquapel treatment. Aquapel was manually injected into the device using a filtered syringe with excess Aquapel flushed out using Novec 7500 oil (3M). The channels were dried by blowing nitrogen and the resultant device was ready to be used for on-chip experiments. For experimentation, the inlet ports of the device were directly connected to oil and aqueous syringes (5 cc BD Luer-Lok Tip, VWR International) fixed on two dual infusion syringe pumps (Harvard Apparatus, Cambridge, MA, USA), to initiate droplet generation.
