# Supplementary material for: FluoroCellTrack: An algorithm for automated analysis of high-throughput droplet microfluidic data
Source: PLoS One. 2019 May 1;14(5):e0215337. doi: 10.1371/journal.pone.0215337 (PMC6493727; doi:10.1371/journal.pone.0215337)
Supplement: S3 Method — (DOCX) [file pone.0215337.s003.docx]

**S3 Method: Experimental Set Up**

*Dose response* *analysis of OPM2 cells to Bortezomib using a droplet trapping array*

OPM2 cells were cultured in three T-25 flasks with a seeding density of 4.5x10^6^ cells/mL. On the day of culture, all flasks were inoculated with 1 nM Bortezomib. Drug efficacy was examined at three timepoints (24 h, 48 h, and 72 h) with each flask being used for a time point. To assess cellular viability at each time point, the entire volume of the flask was supplemented with 2.5 µM Calcein-AM and 4 µM EthD-1 and incubated at 37 °C for 20 min. Calcein AM and EthD-1 are optimal dyes to assess cell viability on fluorescence-based detection platforms: Calcein AM marks live cells and EthD-1 marks dead cells respectively.[1] FITC and rhodamine filter sets were used to observe live and dead cells. Following this incubation period, the cells were transferred into a 5 mL syringe and immediately injected into the microfluidic device. This experimentation procedure was repeated for two additional doses of Bortezomib (5 nM and 10 nM). Control experiments for respective timepoints were also performed in the absence of any drug treatment.

*Tracking single cells using luminescent nanoparticles in a droplet trapping array*

On the day of the experiment, GFP-expressing HeLa cells at a density of 4.5x10^6^ cells/mL were washed with PBS (PBS: 137 mM NaCl, 10 mM Na_2_HPO_4_, 27 mM KCl, and 1.75 mM KH_2_PO_4_ at pH 7.4), detached from flasks via trypsinization, and re-suspended in extracellular buffer (ECB: 20 mM HEPES, 140 mM NaCl, 5 mM KCl, 1 mM MgCl_2_.6H_2_O, 1 mM CaCl_2_.2H_2_O, 5 mM D-Glucose at pH 7.4). The cell suspension was supplemented with rare earth-RE^3+^(Europium:Eu^3+^ and Terbium:Tb^3+^)-doped NPs to give a final NP concentration of 10 mg/mL. These RE-doped NPs work on the basis of downconversion principle,[2] which when upon UV excitation, the RE dopants in NPs exhibited unique emission peaks (Eu^3+^-doped NP in red and Tb^3+^-doped NP in green) that did not overlap with commonly used fluorophores. This slurry of cells and NPs was injected into the microfluidic droplet trapping array to achieve the co-encapsulation of both NPs and cells. A similar approach was performed using RFP-expressing MDA-MB-231 cells. Additionally, the real-time tracking potential of the two luminescent NPs was tested where a mixed population of live and dead MDA-MB-231 cells and NPs was incubated with 4.5 Calcein AM and 2 µM Eth-D1 for 20 min. The FITC and rhodamine filter sets were used to capture GFP expressing cells, live cells and RFP expressing cells, dead cells. Filter set 1 and filter set 2 were used to capture Eu^3+^-doped NPs and Tb^3+^-doped NPs.

*Understanding CPP uptake in intact cancer cells by measuring intracellular fluorescence*

CPPs are peptides that are able to traverse the plasma membrane of intact cells and have been incorporated into biosensors and therapeutics as a novel delivery system.[3] Cell penetrating peptide uptake was quantified in both HeLa and OPM2 cell lines for four different CPPs (novel: RWRWR, OWRWR; commercial: ARG, TAT). On the day of experiment, cells were collected and adjusted to a final density of 4.5 x 10^6^ cells/mL in ECB by centrifugation and re-suspension. The cellular suspension was incubated with two different concentrations of CPPs (10 µM and 50 µM) in microcentrifuge tubes for 60 min at 37°C in the dark. After incubation, the sample was washed twice with PBS to remove any peptide debris and was re-suspended in ECB. The final cell suspension was collected into a 5 mL syringe and injected into the microfluidic droplet trapping array as described above. Intracellular fluorescence was used to assess CPP uptake due to the presence of a 5,6-carboxyfluorescent tag conjugated to all the peptides. Cellular fluorescence was measured using the FITC filter set.

References

1. Ferguson AT. Cellular Imaging Made Easy. Cell. 2018;2018.

2. Gai S, Li C, Yang P, Lin J. Recent Progress in Rare Earth Micro/Nanocrystals: Soft Chemical Synthesis, Luminescent Properties, and Biomedical Applications. Chem Rev. 2014;114(4):2343-89. doi: 10.1021/cr4001594.

3. Wang F, Wang Y, Zhang X, Zhang W, Guo S, Jin F. Recent progress of cell-penetrating peptides as new carriers for intracellular cargo delivery. Journal of Controlled Release. 2014;174:126-36.
