# Supplementary material for: FluoroCellTrack: An algorithm for automated analysis of high-throughput droplet microfluidic data
Source: PLoS One. 2019 May 1;14(5):e0215337. doi: 10.1371/journal.pone.0215337 (PMC6493727; doi:10.1371/journal.pone.0215337)
Supplement: S1 Table — A minimum of 275 cells and n = 787 droplets were analyzed for each case. (DOCX) [file pone.0215337.s004.docx]

**S1 Table. Automated quantification of single OPM-2 cell response to different doses of Bortezomib.** A minimum of 275 cells and n=787 droplets were analyzed for each case.

| **Single OPM-2 cell responses to 1 nM, 10 nM and 100 nM of BTZ** | | | | | | |
| --- | --- | --- | --- | --- | --- | --- |
| **Incubation**  **Time** | **1 nM BTZ** | | | | | |
|  | Single Cell Encapsulation | Multiple Cell Encapsulation | Empty Droplets | Live Cells | Dead Cells | Overlapping Cells |
| Day 1 | 247 | 97 | 443 | 311 | 5 | 28 |
| Day 2 | 80 | 28 | 679 | 53 | 10 | 45 |
| Day 3 | 241 | 158 | 388 | 140 | 53 | 206 |
| **Incubation**  **Time** | **10 nM BTZ** | | | | | |
|  | Single Cell Encapsulation | Multiple Cell Encapsulation | Empty  Droplets | Live Cells | Dead Cells | Overlapping Cells |
| Day 1 | 84 | 8 | 695 | 49 | 5 | 38 |
| Day 2 | 164 | 31 | 592 | 15 | 67 | 113 |
| Day 3 | 187 | 12 | 588 | 0 | 92 | 107 |
| **Incubation**  **Time** | **100 nM BTZ** | | | | | |
|  | Single Cell Encapsulation | Multiple Cell Encapsulation | Empty  Droplets | Live Cells | Dead Cells | Overlapping Cells |
| Day 1 | 140 | 35 | 612 | 0 | 75 | 100 |
| Day 2 | 279 | 193 | 315 | 3 | 320 | 149 |
| Day 3 | 86 | 39 | 662 | 0 | 108 | 17 |
