# Supplementary material for: FluoroCellTrack: An algorithm for automated analysis of high-throughput droplet microfluidic data
Source: PLoS One. 2019 May 1;14(5):e0215337. doi: 10.1371/journal.pone.0215337 (PMC6493727; doi:10.1371/journal.pone.0215337)
Supplement: S2 Table — Information on droplet subpopulations and cell encapsulation obtained from two individual experiments where RFP-expressing MDA-MB-231 cells were co-encapsulated with Eu3+-doped NPs and Tb3+-doped NPs. n = 787 droplets were analyzed in each case. (DOCX) [file pone.0215337.s005.docx]

**S2 Table. Quantification of droplet tracking data by FluoroCellTrack.** Information on droplet subpopulations and cell encapsulation obtained from two individual experiments where RFP-expressing MDA-MB-231 cells were co-encapsulated with Eu^3+^-doped NPs and Tb^3+^-doped NPs. n=787 droplets were analyzed in each case.

| **Droplet tracking with luminescent nanoparticles** | | | | | |
| --- | --- | --- | --- | --- | --- |
| **RFP-expressing MDA-MB-231 cells tracked by Eu^3+^-NPs** | | | | | |
| Single Cells tracked by NP | Multiple Cells tracked by NP | Droplets  with only NP | Droplets with only single cells | Droplets with only multiple cells | Droplets with no NP and cells |
| 238 | 161 | 354 | 14 | 9 | 11 |
| 215 | 153 | 386 | 7 | 14 | 12 |
| **RFP-expressing MDA-MB-231 cells tracked by Tb^3+^-NPs** | | | | | |
| Single Cells tracked by NP | Multiple Cells tracked by NP | Droplets  with only NP | Droplets with only single cells | Droplets with only multiple cells | Droplets with no NP and cells |
| 195 | 231 | 305 | 18 | 13 | 25 |
| 248 | 186 | 320 | 11 | 9 | 13 |
