# Supplementary material for: FluoroCellTrack: An algorithm for automated analysis of high-throughput droplet microfluidic data
Source: PLoS One. 2019 May 1;14(5):e0215337. doi: 10.1371/journal.pone.0215337 (PMC6493727; doi:10.1371/journal.pone.0215337)
Supplement: S3 Table — A minimum of 142 cells (maximum 367 cells) were analyzed in these experiments. (DOCX) [file pone.0215337.s006.docx]

**S3 Table.** **Comparison of mean intracellular fluorescence representative of CPP uptake in HeLa cells using FluoroCellTrack.** A minimum of 142 cells (maximum 367 cells) were analyzed in these experiments.

| **Quantification of intracellular fluorescence in HeLa cells – mean fluorescence (AU)** | | | |
| --- | --- | --- | --- |
| **CPP** | **FluoroCellTrack** | **Manual Analysis** | **Difference**  **(%)** |
| 50 µM ARG | 12791 | 12462 | 2.65 |
| 50 µM TAT | 2849 | 2617 | 8.80 |
| 50 µM RWRWR | 5064 | 4877 | 3.83 |
| 50 µM OWRWR | 5178 | 4963 | 4.33 |
